# Apomixis frequency under stress conditions in weeping lovegrass (*Eragrostis curvula*)

**DOI:** 10.1371/journal.pone.0175852

**Published:** 2017-04-18

**Authors:** Juan Manuel Rodrigo, Diego Carlos Zappacosta, Juan Pablo Selva, Ingrid Garbus, Emidio Albertini, Viviana Echenique

**Affiliations:** 1 CERZOS-CONICET, CCT-Bahía Blanca, Bahía Blanca, Argentina; 2 Departamento de Agronomía, Universidad Nacional del Sur, Bahía Blanca, Argentina; 3 Departamento de Biología, Bioquímica y Farmacia, Universidad Nacional del Sur, Bahía Blanca, Argentina; 4 Departamento de Ciencias de la Salud, Universidad Nacional del Sur, Bahía Blanca, Argentina; 5 Department of Agricultural, Food and Environmental Science, University of Perugia, Perugia, Italy; Georg-August-Universitat Gottingen, GERMANY

## Abstract

To overcome environmental stress, plants develop physiological responses that are triggered by genetic or epigenetic changes, some of which involve DNA methylation. It has been proposed that apomixis, the formation of asexual seeds without meiosis, occurs through the temporal or spatial deregulation of the sexual process mediated by genetic and epigenetic factors influenced by the environment. Here, we explored whether there was a link between the occurrence of apomixis and various factors that generate stress, including drought stress, *in vitro* culture, and intraspecific hybridization. For this purpose, we monitored the embryo sacs of different weeping lovegrass (*Eragrostis curvula* [Schrad.] Nees) genotypes after the plants were subjected to these stress conditions. Progeny tests based on molecular markers and genome methylation status were analyzed following the stress treatment. When grown in the greenhouse, the cultivar Tanganyika INTA generated less than 2% of its progeny by sexual reproduction. Plants of this cultivar subjected to different stresses showed an increase of sexual embryo sacs, demonstrating an increased expression of sexuality compared to control plants. Plants of the cv. Tanganyika USDA did not demonstrate the ability to generate sexual embryo sacs under any conditions and is therefore classified as a fully apomictic cultivar. We found that this change in the prevalence of sexuality was correlated with genetic and epigenetic changes analyzed by MSAP and AFLPs profiles. Our results demonstrate that different stress conditions can alter the expression of sexual reproduction in facultative tetraploid apomictic cultivars and when the stress stops the reproductive mode shift back to the apomixis original level. These data together with previous observations allow us to generate a hypothetical model of the regulation of apomixis in weeping lovegrass in which the genetic/s region/s that condition apomixis, is/are affected by ploidy, and is/are subjected to epigenetic control.

## Introduction

Apomixis is a form of asexual seed production that avoids both meiosis and fertilization. In the apomictic pathway, differentiated MMCs, or somatic cells within ovules that acquire a germinal cell fate, are able to either entirely bypass meiosis or undergo abnormal meiosis to produce unreduced spores that then divide mitotically to form an embryo sac [1, 2]. Although apomixis is genetically regulated and occurs naturally in more than 120 angiosperm genera that belong to approximately 40 families [2], its underpinnings at the molecular level are still unclear. Some genes [3] or genomic regions [4] have been associated with the trait. Most apomictic plants exhibit sexual reproduction to some degree and are facultative apomicts [5]. True obligate apomicts (individuals that produce only asexual embryo sacs) are uncommon. Apomixis may represent a strategy to overcome problems in development caused by cytological abnormalities. Carman [2] postulated that such abnormalities can be produced by asynchronous and ectopic expression of several developmental pathways determined by multiple genomes in polyploid plants. In addition, there is increasing evidence that epigenetics may regulate apomixis [6].

In organisms that can produce both sexually and asexually, stress plays an important role in determining which reproductive mode is used [7]. Different species of fungi, including *Saccharomyces cerevisiae*, *Neurospora crassa*, and *Candida albicans*, induce sexual sporulation under low nitrogen conditions [8, 9]. In the algae *Volvox carteri* [10] and *Chlamydomonas reinhardtii* [11] sexual reproduction is triggered in response to abiotic stress. The reproductive modes of grasses also respond to environmental conditions; for instance *Themeda australis* increases apospory during short days [12], *Dichanthium aristatum* shows a quantitative change in the incidence of apomixis in response to changes in photoperiod [13] and *Ranunculus auricomus* increase sexuality under long photoperiod [14]. Moreover, Gounaris et al [15] detected a greater number of reduced embryo sacs under salt stress in *Cenchrus ciliaris*.

Examples of induction of sexual reproduction in response to stress are not confined to the fungal and plant kingdoms. Water fleas (*Daphnia pulex*) usually reproduce parthenogenically between spring and late summer, producing only females. During winter or periods of drought, males are also produced and the population begins to reproduce sexually [16]. These examples make it clear that stress-related cues induce the switch from asexual to sexual reproduction in a range of organisms. Based on this, it is reasonable to expect that facultative apomicts tend to switch to sexual reproduction more often under stress conditions, and that such stress-dependent switch facilitates the organism’s adaptation to a stressful environment.

Recent studies on the regulation of reproductive mode indicate that female gametogenesis is regulated by epigenetic mechanisms that are crucial for distinguishing sexual from apomictic development [6, 17]. Furthermore, growing evidence suggests that methylation of DNA in response to stress affects gene regulation, leading to phenotypic variation, providing a mechanistic link between stress and apomixis [18].

Weeping lovegrass (*Eragrostis curvula* [Schrad.] Nees) is a perennial grass native to Southern Africa that displays a type of apomixis termed pseudogamous diplospory [19]. The *E*. *curvula* complex includes cytotypes with different ploidy levels (from 2x to 8x) that may undergo sexual reproduction, facultative apomixis, or obligate apomixis [20]. Diploid (2n = 2x = 20) *E*. *curvula* plants are sexual and rare [21]. *E*. *curvula* polyploids are mostly obligate apomicts, although both sexual reproduction and facultative apomixis have also been reported [20]. Previously, we found some evidence for a change in the frequency of apomixis in this grass under stress situations such as during *in vitro* culture and polyploidization [22].

Here, we studied the effect of different factors that generate stress (water deficit, *in vitro* culture, and intraspecific hybridization) on the frequency of apomixis in the diplosporous weeping lovegrass. Cytoembryological studies and progeny tests were carried out in plants of different genotypes subjected to stress conditions, and the genome methylation level was analyzed during and after different stress treatments.

## Materials and methods

### Plant material

For the study we used the following apomictic tetraploid (2*n* = 4*x* = 40) cultivars: Don Walter (DW) and two accessions of Tanganyika (TI and TU). The DW and TI accessions were provided by INTA (Instituto Nacional de Tecnología Agropecuaria, Argentina) and TU was from the USDA (accession PI234217, United States Department of Agriculture). Other plant material used in this study was the sexual tetraploid OTA-S (accession PI574506 USDA). Plants were cultivated in 10-l pots under greenhouse conditions with the natural photoperiod of approx. 15 h at spring flowering period (Bahía Blanca, Buenos Aires, Argentina; 38° 42´ S, 62° 16´ W).

### Treatments

#### Drought stress

TI and TU plants (four plants each) were exposed to drought stress conditions beginning three months before the onset of flowering and lasting until the end of the flowering season (September to March, 2010–2011), with a weekly watering of 50–80 ml per pot. Plants of the same cultivars at the same developmental stage and maintained in optimum irrigation conditions (300–500 ml/week) were used as controls (four plants each). Relative water content (RWC) of plants was determined on leaves using the following formula: RWC (%) = [(FM—DM)/(TM—DM)] x 100, where, FM, DM, and TM are the fresh, dry, and turgid tissue weight, respectively [23]. The same treatment was applied to DW plants (four plants under each treatment) during the flowering season 2015–2016.

#### *In vitro* culture

Callus induction and plant regeneration were conducted using TI and TU seeds as explants, following the protocol of Echenique et al. [24]. After tissue culture, the regenerated plants were carefully transferred to greenhouse conditions and cultivated for six months. Plants at the same development stage from the same cultivars germinated in pots and grown in the greenhouse under the same conditions were used as controls.

#### Intraspecific hybridization

Six hybrids, obtained in 2010 from controlled crosses between the sexual genotype OTA-S and the apomictic cultivar TU, were evaluated in three flowering seasons.

### Cytoembryological studies

To analyze the effect of stress on apomixis-sexual relationship, we study the mode of reproduction at two developmental stages, early at megasporogesis and megagametogenesis and at the seed stage (progeny test). In the former case, inflorescences from plants subjected to the described treatments were collected at the beginning of anthesis, when all embryo sac developmental stages are observable, and were fixed in FAA (50% ethanol, 5% acetic acid, 10% formaldehyde in distilled water). Individual spikelets were dehydrated in a tertiary butyl alcohol series and embedded in Paraplast (Leyca Paraplast Plus, USA) [25]. Samples were sectioned at 10 μm and stained with safranin-fast green. Observations were carried out with a Nikon Eclipse TE300 light transmission microscope (Tokyo, Japan). The mode of reproduction was assessed taking into account the presence of meiotic processes or the number and position of nuclei during different stages of the embryo sac development [26].

### Progeny tests

Progeny tests were performed on seeds obtained by open pollination from control and treated plants. Progeny tests comprised 40 to 45 plants and were performed in a similar manner as was previously reported by Matzk et al [27]. Genomic DNA was extracted from fresh leaf tissue following the procedure described by Meier et al [26]. RAPD reactions were performed using the protocol described at CIMMYT (http://www.cimmyt.org) with four primers from the NAPS unit list of standard primers (S1 Table). Amplification products were electrophoresed in 6% (w/v) acrylamide gels and silver stained. Offspring plants were classified as apomictic when RAPD-derived genetic profiles were identical or exhibited only one polymorphism with respect to the maternal profile. They were classified as sexual only when two or more polymorphisms with respect to the maternal were observed, to avoid errors due to experimental artifacts [28].

### Amplified Fragment Length Polymorphisms (AFLPs)

AFLP studies were performed according to Vos et al. [29] on DNA samples extracted from leaves of cultivar TI. Genomic DNA samples (600 ng) were double digested with the enzymes *Pst*I and *Mse*I. The resulting fragments were then ligated to *Pst*I and *Mse*I adaptors to produce templates for further amplifications. PCR amplifications using technical duplicates were carried out with seven AFLP primer combinations and inconsistent bands were not taken into account (S1 Table). PCR products were separated on 6% (w/v) denaturing polyacrylamide gels, silver-stained, and digitized for analysis.

### Methylation-Sensitive Amplified Polymorphisms (MSAPs)

Modifications of the cytosine methylation pattern were detected by performing MSAP studies on DNA samples extracted from leaves and panicles according to Xu et al. [30]. In the case of cultivar TI DNA samples were extracted from leaves and in TU and Don Walter DNA was taken from panicles. The same DNA used in the AFLP studies was used to compare plants at the same sampling times. The methylation-sensitive isoschizomers *Hpa*II and *Msp*I were selected as frequent-cutting enzymes, and *Eco*RI was chosen as a rare-cutting enzyme. PCR amplifications were carried out with seven MSAP primer combinations for the comparisons between during and after stress, using technical duplicates (S1 Table). PCR products were separated in 6% (w/v) denaturing polyacrylamide gels, silver-stained, and digitized for analysis. Bands were counted only when they were present in both replicates. Variation of less than 5% between technical replicates was required for a primer combination to be included in the analysis. The interpretation of MSAP data is based on known restriction enzyme activities at recognition sequences modified by methylation. For the polymorphisms classification as methylation or demethylation, we followed the criteria proposed by Fulneček and Kovařík [31] that is explained in S2 Table. These authors propose that different methylated forms may provide identical MspI/HpaII digestion profiles, in these cases this pattern was named ambiguous polymorphisms and are discarded from the analyses.

### Isolation, cloning, and sequencing polymorphic DNA fragments

Bands were moistened with distilled sterile water and excised from polyacrylamide gels using a scalpel. They were then cut into smaller sections and eluted with buffer solution (0.5 M ammonium acetate, 1 mM EDTA pH 8) overnight at 37°C. The DNA was ethanol-precipitated and re-amplified using the same PCR conditions described for AFLP and MSAP assays. The resulting fragments were cloned using the pGEM^®^-T

### Sequence data analysis

Sequencing of the differential MSAP fragments was performed at Ibiotec (Institute of Biotechnology, INTA Castelar, Argentina). Homology searches were conducted using the algorithm BLASTX against the nonredundant protein database (nr) and BLASTN against the nt (nucleotide collection) deposited at NCBI (NCBI, http://www.ncbi.nlm.nih.gov/; version 2.2.29) to assign putative functions to the sequenced fragments. For BLASTX and BLASTN analyses, the result with the lowest e-value and the highest similarity score was considered the best match for the putative identity of the corresponding protein or nucleotide sequence, with a minimum threshold e-value of e^-05^. The sequence data generated herein is available in the dbEST database.

### Statistical analysis

Student's t-test was used to analyze the results of cytoembryology and progeny tests. A paired t-test was used to compare the rehydrated plants in time. The relationships between sexual reproduction and methylation changes over time were evaluated by the parametric Pearson correlation. All statistical analyses were performed using Infostat software version 2010 (Infostat computational pack http://www.infostat.com.ar, FCA-UNC, Argentina).

## Results and discussion

### Assessing the level of sexual reproduction in the apomictic cv. Tanganyika

**Fig 1:**
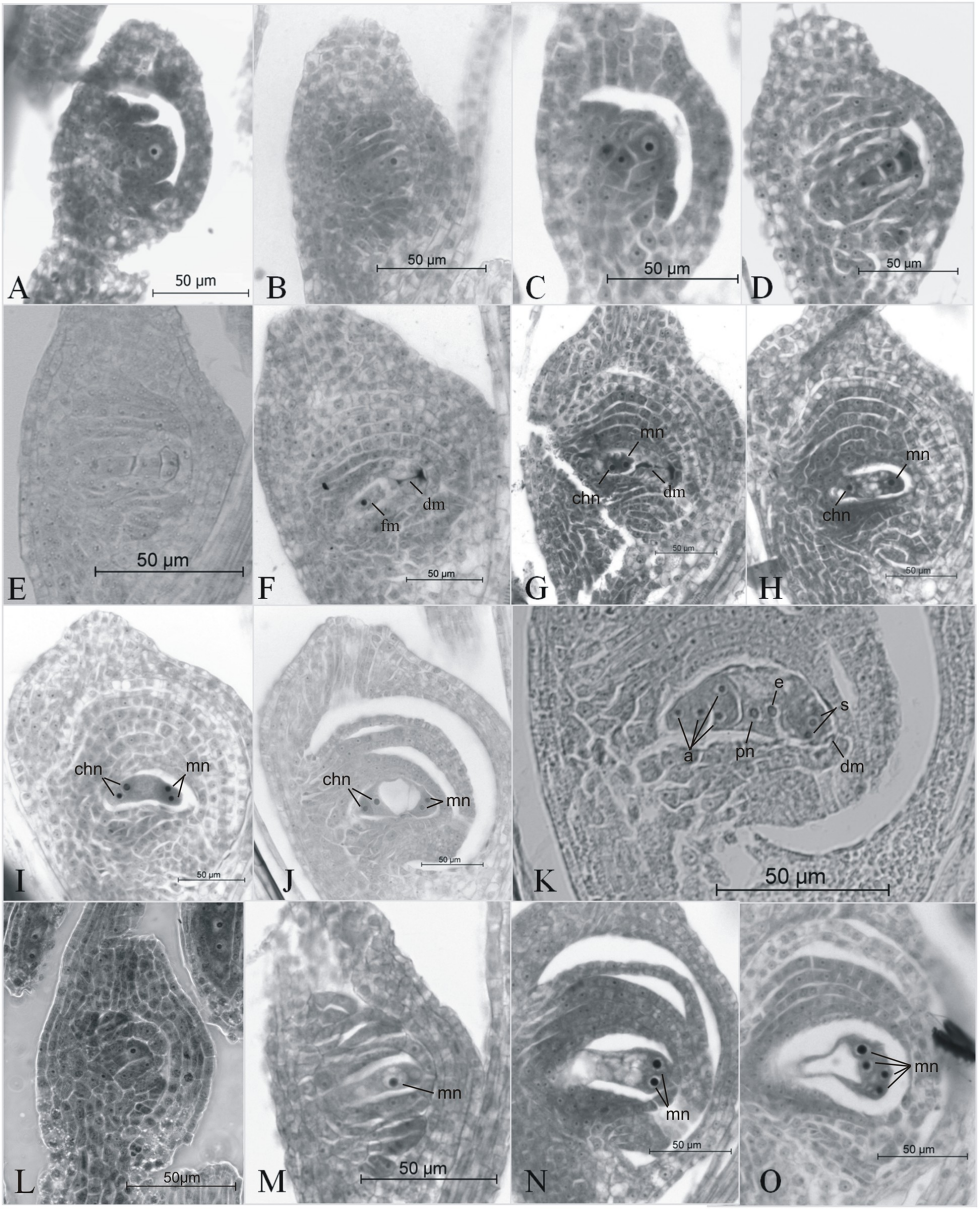
Sexual (A-K) and diplosporous (L-O) embryo sac development in weeping lovegrass cv. Tanganyika INTA plants. Sections stained with safranin-fast green. (A) Archesporial cell. (B) Megaspore mother cell. (C) First meiotic cell division. (D) Three cells of the linear tetrad. (E) Linear tetrad of megaspores. (F) Functional chalazal megaspore and degenerated megaspores. (G) Binucleated stage and degenerated megaspores. (H) Advanced binucleated stage. (I) Tetranucleated stage. (J) Advanced tetranucleated stage. (K) Octanucleated stage. (L) Differentiation of the megaspore mother cell. (M) Elongated megaspore mother cell. (N) Binucleated stage. (O) Tetranucleated stage. a: antipodal, chn: chalazal nucleus, dm: degenerated megaspore, e: egg, fm: functional megaspore, mn: micropilar nucleus, pn: polar nucleus, s: synergid.

To assess the normal ratio of sexual to apomictic embryo sacs during the flowering period in weeping lovegrass (Fig 1), we analyzed more than 1000 pistils in the optimal developmental stage (the beginning of anthesis) in cv. Tanganyika INTA plants (TI) growing under stable conditions in the greenhouse over two consecutive years. The sexual megasporocyte of *E*. *curvula* undergoes meiosis and three rounds of mitotic divisions to form a reduced octonucleate embryo sac with an egg, two synergids, two polar nuclei and three antipodals. By the other hand the apomictic megasporocyte undergoes two rounds of mitotic divisions to form a non-reduced tetranucleate embryo sac with an egg, two synergids, and one polar nucleus [26]. We can observe common stages for apomictic and sexual pistils, like archesporial cell (Fig 1A) and megaspore mother cell (Fig 1B and 1L) and typical stages indicative of sexual processes, such as first meiotic cell division (Fig 1C), three cells of the linear tetrad (Fig 1D), linear tetrad of megaspores (Fig 1E), functional and degenerated megaspores (Fig 1F), binucleated embryo sac (Fig 1G and 1H), tetranucleated embryo sac (Fig 1I and 1J) and octanucleated embryo sac (Fig 1K). In the other hands, typical apomictic processes were observed like elongated megaspore mother cell (Fig 1M), binucleated embryo sac (Fig 1N) and tetranucleated embryo sac (Fig 1O). In the current study the way to distinguish between both categories, sexual or apomictic in the cytoembryological analyses is to consider, in the case of sexual processes, the stages present in Fig 1C to 1K. These stages comprise from meiosis to mature octonucleated embryo sac. In the case of apomictic processes we considered the stages showed in Fig 1M to 1O, comprising from elongated megaspore mother cell to mature tetranucleated embryo sac.

We determined that, under our controlled conditions, the level of sexual reproduction of TI plants averaged 2.4% (ranging between 2.3% and 2.5%, depending on the blooming period). These findings demonstrate that the apomictic cultivar TI harbors a stable residual sexuality. However, we cannot rule out some degree of seasonal variation in the level of sexuality in *E*. *curvula* plants growing under field conditions. *Paspalum notatum*, other apomictic Poaceae, exhibits a lower frequency of sexuality in the middle of the flowering season [32]. This low level of background sexuality is not present in all weeping lovegrass cultivars, as the cultivar TU does not display sexual reproduction under controlled conditions at all [26].

### Expression of sexuality in drought-stressed plants of the cv. Tanganyika and Don Walter

**Fig 2:**
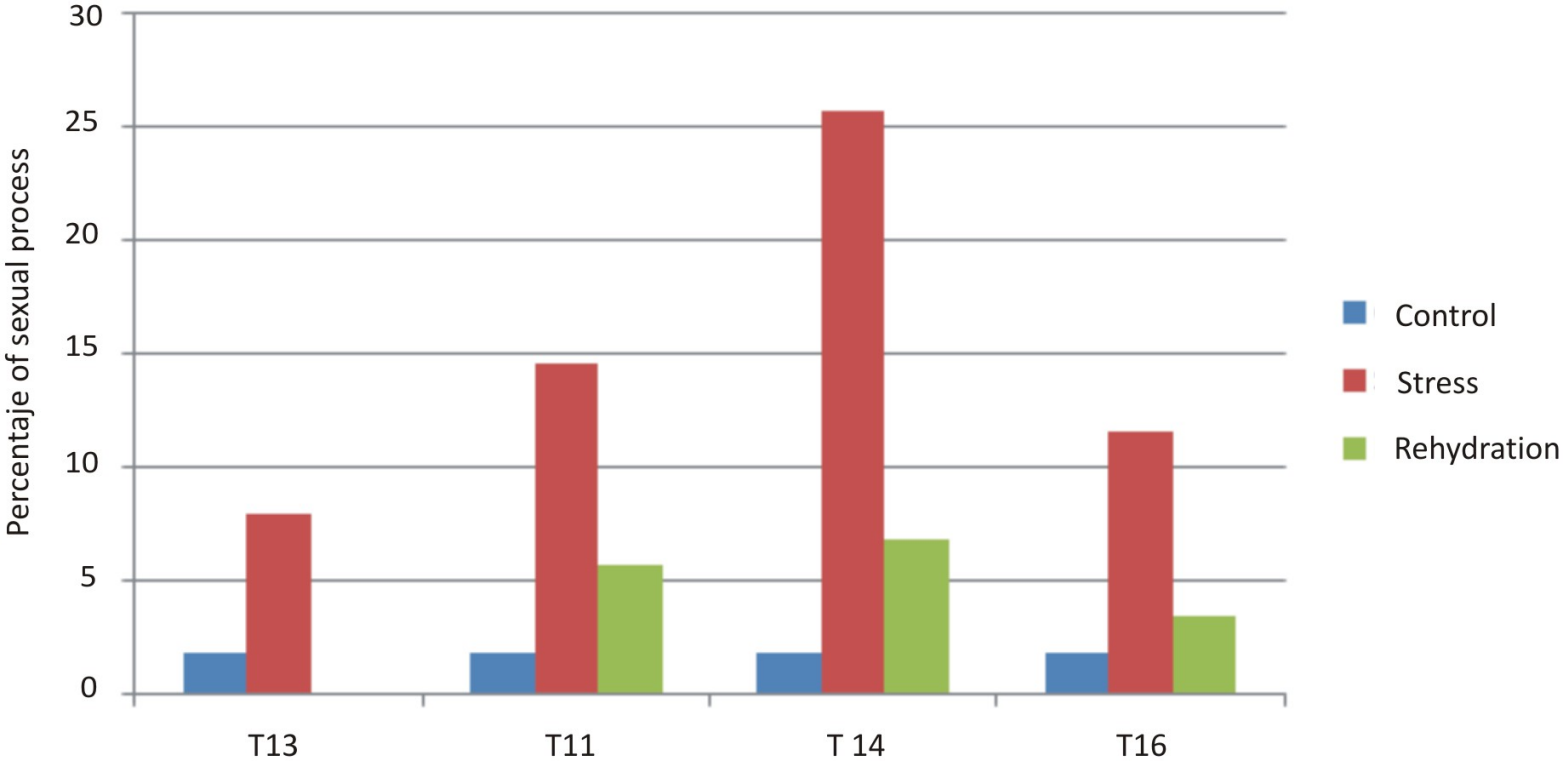
Effect of drought stress treatment. Percentage of sexual processes in plants belonging to the cv. Tanganyika INTA (TI) during drought stress conditions and after the rehydration treatment. Control represents the average of sexual processes observed in control plants. TI13 plant at rehydration moment didn´t flowering.

The number of pistils and the ratio of sexual to apomictic embryo sacs from control and drought-stressed TI plants are shown in Fig 2. In control plants, with an average Relative water content (RWC) of 89%, 1.8% of embryo sacs were sexual (7 of 398), as measured by cytoembryological studies, a difference that is not statistically significant from our previously measured baseline of 2.4%. Under drought-stress conditions, with an average RWC of 49.7%, the percentage of sexual embryo sacs increased dramatically to 14.4% (47 of 327), significantly different from the corresponding value of control plants (p ≤ 0.05).

The same drought stress treatment was applied to plants of another apomictic facultative cultivar, Don Walter. In this case, there was a statistically significant increase in the percentage of sexual processes from 4% (n = 85) in the control plants to 22% (n = 149) in the water stressed ones.

Other authors have also reported variation in the frequency of apomixis caused by different abiotic factors [12, 13, 33]. Gounaris et al. [15] showed that plants of the aposporous buffelgrass (*Cenchrus ciliaris*) grown under high salt conditions *in vitro* produced a higher proportion of sexual embryo sacs than controls. The authors concluded that the low cellular water content induced by the high salt concentration probably affects cell growth, cell differentiation, and development. Carman [34], working with two taxa of *Boechera*, showed that plants growing under drought and heat stress conditions showed a two to three fold increase in the number of sexual tetrads.

After the drought-stress period, plants were rehydrated and maintained in optimal conditions beginning three months before the next flowering period to assess the frequency of sexual reproduction after recovery from the stress. Inflorescences were collected and the numbers of apomictic and sexual embryo sacs were recorded (Fig 2). The average RWC of the treated plants was similar to that of control plants (average of 89%). The rehydrated plants showed a 5.3% frequency of sexual embryo sacs (18 of 341), indicating that sexual reproduction decreased significantly (p ≤ 0.05) after rehydration compared to during the stress. Nevertheless, the frequency of sexual reproduction remained significantly higher than in control plants (p ≤ 0.05).

Although these results suggest that the increased frequency of sexual reproduction could increase genetic variation in response to stress, the progeny tests indicated a lower level of variation than expected based on the number of sexually produced embryo sacs. This suggests that not all of the sexual embryo sacs led to viable plants. Using RAPD markers on the offspring of three TI control plants, we were unable to detect any polymorphisms relative to the maternal plant. In drought-stressed plants, the same test using 245 offspring (60 offspring per each plant) produced polymorphic bands less frequently (0–5% of individuals) than expected based on the cytoembryological observations (14.4% of sexual embryo sacs). Only in the two out four treated plants sexual processes were observed (4.6 and 2.5% of variable progeny respectively), while in the other two plants variable progeny was not observed. However, even this low level of polymorphism per plant would generate a large amount of variation at the population level, as each plant produces a very large number of seeds. Similar results were obtained by Rebozzio et al [32] in *P*. *notatum* and by Carman [34] in *Boechera*; in *Boechera*, most ovules fail to produce seeds under stress conditions and most seeds are formed apomictically. Moreover, Hojsgaard et al [35] demonstrated that aposporous embryo sacs develop faster than sexual ones, as they skip meiosis. Thus, the key factor that leads to the reduction in the number of sexually produced progeny would be the faster growth of embryos derived by the aposporous pathway [35]. In aposporous plants, apomixis and sexuality can coexist and compete in the same egg, generally reducing the frequency of sexually derived progeny [35]. In diplosporous plants, residual sexuality is less frequent, as the archespore cell must choose one developmental pathway. In many cases, this results in the need to evaluate a large number of seeds to find any generated by sexual reproduction [36]. In general, however, these sexual events produce enough genetic variability in natural populations to respond to environmental changes [37]. Houliston et al [33], working with *Hieracium pilosella*, reported that sexual events, although they constitute only between 0.2 to 6% of the progeny, are important for responding to environmental change. Apomixis is often an escape from sexual sterility—in triploids for example, in many odd-level polyploids, in interspecific diploid hybrids (which by definition are sexually sterile), as in *Boechera*, and in autopolyploids (which often show reduced levels of fertility due to unbalanced gamete formation) [38].

When the same stress treatment by water deprivation was applied to TU plants, we were unable to detect the presence of sexual embryo sacs despite the analysis of more than 1000 pistils in both control and treated plants (RWC 83% and 49%, respectively), suggesting that plants of this cultivar are true obligate apomicts.

### *In vitro* culture treatment

Tissue culture of plants is an important means to propagate genetically identical individuals asexually. However, undesired genetic and cytogenetic modifications are frequently induced during tissue culture. Although tissue culture-induced mutations have been studied extensively as a source of plant improvement, little is known about their molecular causes. Mutations or epigenetic changes induced during *in vitro* culture could be responsible for this effect [39].

We conducted *in vitro* culture using TI seeds as explants and maintained the callus stage for five or eight months to obtain 23 plants. After two years in the greenhouse, only one of these plants produced a small inflorescence. Cytoembryological analysis of all pistils of this inflorescence showed a frequency of sexual reproduction of 33.3% (22 of 66). This result indicates that certain tissue culture conditions can induce the expression of sexual reproduction, and demonstrates that tissue culture alters normal reproductive development in *E*. *curvula*. Mutations or epigenetic changes induced during *in vitro* culture could be responsible for this effect.

When explants from TU plants were used, we could regenerate plants only from calli up to five months old. After this period, the calli lost their morphogenetic capacity. Cytoembryological analysis of inflorescences of plants derived from five-month-old calli showed only apomictic embryo sacs, providing further evidence of the obligate apomictic nature of this cultivar.

### Intraspecific hybridization

From the nearly three thousand seeds obtained from crosses between OTA-S x TU, only six hybrid plants were obtained. We analyzed these plants in their first flowering season (2010) and classified four plants as sexual and two as apomictic (the presence of apomictic processes is sufficient to classify the plant as such). Of the two apomictic plants, one of them generated 46.2% (#39) diplosporous embryo sacs and the other 85.6% (#105).

In the next flowering season (2011/12) only two out of these six plants survived, one sexual (#60) and one apomictic (#105). Cytoembryological analysis of these plants in the first blooming period (September—December) showed frequencies of sexual to apomictic embryo sacs similar to those of the previous year. In the second blooming period (January—March), hybrid #60 present only sexual ovary and hybrid #105 exhibited a large decrease in the percentage of apomictic embryo sacs (from 88% to 29.7%). In the next flowering season (2012/13), hybrid #105 showed a 45–65% frequency of apomictic reproduction. Surprisingly, in the same period the previously sexual hybrid #60 produced three diplosporous embryo sacs (3 of 68 analyzed pistils).

The instability of the frequency of apomixis in the hybrid #105 could be due to stress produced by the interaction of divergent parental genomes. It was previously observed that hybridization can lead to genetic rearrangements and epigenetic changes [40]. Although we are working with two cultivars of *E*. *curvula*, the low number of hybrids we obtained is indicative of some kind of incompatibility. One possibility is that, after the stress generated by hybridization, the hybrid genomes are still in a state of reorganization or adaptation that also affects the frequency of apomixis. The analysis of inflorescences from different seasons in hybrid #105 showed that the frequency of apomixis vs. sexual reproduction remained erratic and close to 50%. The observation of apomictic embryo sacs in hybrid #60, which was determined to be sexual during the first evaluation year, demonstrates that it is necessary to evaluate hybrids for more than one year to assess their mode of reproduction.

Aliyu et al [36] evaluated 71 accessions of the genus *Boechera*, and found examples ranging from fully sexual to fully apomictic. Facultative apomictic species showed a strong bimodal distribution, with either a mostly sexual or mostly apomictic mode of reproduction. When comparing the mode of reproduction with the phylogenetic origin of the species, the authors hypothesized that low levels of apomeiosis represents the ancestral condition in *Boechera*, and high levels of apomeiosis can be induced by changes in the global regulation of genes associated with hybridization. Based on this hypothesis, we would expect a high level of apomixis in our weeping lovegrass hybrids. Future studies in our two remaining hybrids and the generation of a larger hybrid population will be needed to determine if this behavior is maintained over time, stabilized with a clear tendency towards a particular mode of reproduction, or remains variable due to unknown mechanisms.

Progeny testing of hybrid #105 using RAPD markers showed that eight of 37 offspring plants (21.5%) showed polymorphic bands. Thus, the fraction of progeny derived from sexual reproduction was lower than that detected by cytoembryology, again demonstrating that not all sexually derived embryo sacs produce viable plants.

### Genome methylation and polymorphic DNA fragments

Since the alterations observed under different stress situations could be due to genetic or epigenetic alteration, we analyzed AFLP and MSAP marker profiles to assess the effects of stress at the genetic and epigenetic levels, respectively. The following comparisons were carried out: I) DNA from drought-stressed (four individuals) and control (two individuals) TI plants obtained from leaf samples collected during the stress and after the re-hydration period and II) DNA of hybrids #60 and #105 extracted in 2010 (first year of analysis) and after three years (2013).

In control and drought-stressed TI plants, seven AFLP primer combinations (M31-P36, M31-P37, M31-P40, M39-P36, M39-P37, M39-P40 and M43-P40) yielded a total of 294 monomorphic markers. Thus, we did not detect any genetic changes among the clonal plants during and after the drought-stress treatment.

Methylation profiles of control and drought-stressed TI plants were analyzed using six MSAP primer combinations (HM6-E37, HM6-E40, HM4-E32, HM4-E37, HM4-E40, HM7-E37; HM7-E40). Comparisons of each individual plant revealed a high number of polymorphic markers between the two sampling times, due to DNA methylation and demethylation. Some of the markers did not show a clear correspondence to methylation or demethylation changes and, according to Fulneček and Kovařík [31] were classify as ambiguous polymorphism (Table 1). Stressed plants (TI11, TI13, TI14, and TI16) showed a higher number of methylation changes than control plants (TI04 and TI20). At individual plant level, it was possible to detect a global but slight trend to methylation in stressed plants and to demethylation in control ones. Only 6% of the MSAP polymorphisms were present in all the individuals, leading us to conclude that most of the methylation changes occurred in a random fashion.

**Table 1:** Variation in MSAP profiles in drought stressed TI plants. Drought stressed (TI11, TI13 TI14 and TI16) and control (TI04 and TI20) plants of cv. Tanganyika INTA. Comparison of MSAP profiles between samples collected during the stress and after the re-hydration period.

MSAP profile	TI11		TI13		TI14		TI16		TI04		TI20	
No change over time	320	87.9%	346	95%	317	87.1%	339	93.1%	350	96.2%	354	97.3%
Methylations	11	3%	5	1.4%	11	3%	7	1.9%	1	0.3%	1	0.3%
Demethylations	8	2.2%	5	1.4%	9	2.5%	6	1.6%	3	0.8%	2	0.5%
Ambiguous	25	6.9%	8	2.2%	27	7.4%	12	3.3%	10	2.7%	7	1.9%

**Fig 3:**
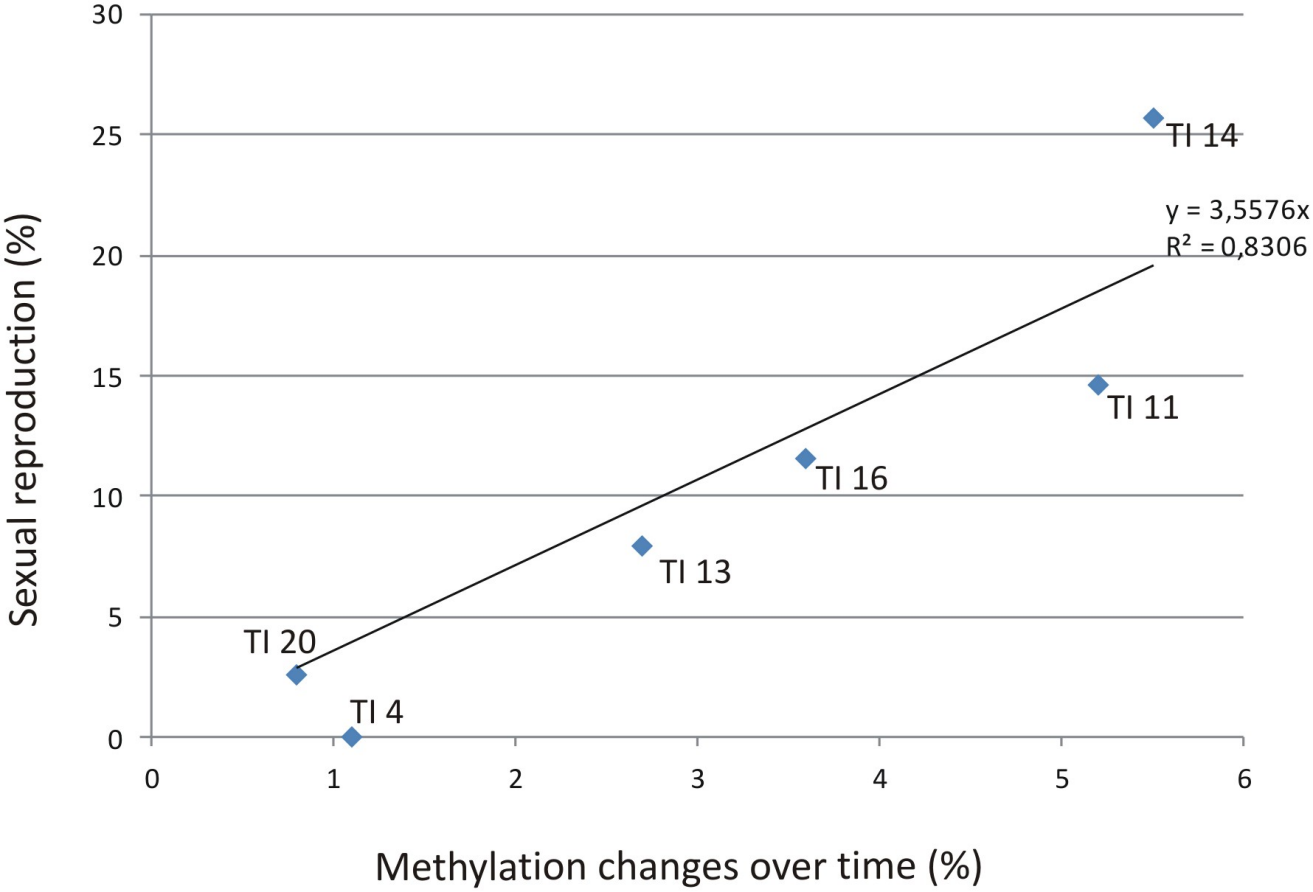
Methylation vs. sexual reproduction. Correlation between methylation changes and the percentage of sexual reproduction in control and stressed plants of weeping lovegrass. Samples from stressed plants was taken during and after treatment.

When the total number of methylation changes was compared between stressed and control plants significant differences were detected and a strong correlation (R^2^ = 0.83) (Fig 3) between methylation changes over time (Table 1) and the frequency of sexual reproduction after treatment was observed (Fig 2).

The MSAP analysis using fifteen primer combinations (HM8-E36, HM8-E41, HM8-E40, HM9-E36, HM9-E41, HM9-E40, HM10-E36, HM10-E41, HM10-E40, HM11-E36, HM11-E41, HM11-E40, HM3-E42, HM3-E43, HM2-E43) on DNA from panicles of control and drought stressed Don Walter INTA plants showed a similar trend to the one observed in cultivar TI. Comparisons between stressed plants revealed a lower amount of polymorphic markers in comparison with TI stressed plants (Table 2) and a similar general behavior with a slight tendency to methylation. A similar situation was observed in the TU cultivar, where stressed plants showed 13 polymorphic bands compared with the control ones (4 methylations, 3 demethylation changes and 6 ambiguous polymorphisms).

**Table 2:** Variation in MSAP profiles in drought stressed DW plants. Polymorphism between drought stressed and control plants of cv. Don Walter INTA.

MSAP profile	DW19		DW11		DW01		DW16	
No change	1070	99%	1042	96.5%	1038	96.1%	1055	97.7%
Methylations	4	0.4%	8	0.7%	13	12%	5	0.5%
Demethylations	0	0%	5	0.5%	8	0.7%	7	0.6%
Ambiguous	6	0.6%	25	2.3%	21	1.9%	13	1.2%

Some authors have speculated that the persistence and ecological success of some asexual lineages is due to epigenetic variability, with this variability serving as a source of phenotypic plasticity and heritable variation [41, 42]. Our analysis suggests the existence of a relationship between stress, epigenetic changes and an increase in the frequency of sexual reproduction in *E*. *curvula*, providing the species an advantage to perpetuate itself in critical environmental situations.

To compare this epigenetic response with another source of stress, in this case the genomic stress caused by hybridization, the genetic and epigenetic structure of the sexual hybrid #60 and the apomictic hybrid #105 was analyzed in 2010 and three years after hybridization (in 2013) using AFLP and MSAP markers. Seven AFLP primer combinations (P40-M39, P40-M43, P40-M45, P37-M38, P37-M39, P37-M43, and P37-M45) revealed only monomorphic markers (243) in hybrid #60, whereas hybrid #105 showed 295 monomorphic and 16 polymorphic markers (during this time period, ten new bands appeared and six bands disappeared). Hybrid #105 therefore displayed more genetic changes, together with a much higher variability in the frequency of sexual reproduction, than hybrid #60, which also showed more stability in its mode of reproduction (between 97 and 100% of sexual embryo sacs) over the period analyzed. At the epigenetic level, a total of 254 and 287 MSAP markers were identified with six primer combinations (HM6-E32, HM6-E37, HM6-E40, HM7-E32, HM7-E37, and HM7-E40) in hybrids #60 and #105, respectively. The vast majority (92% and 94%) of these markers were monomorphic over time (Table 3).

**Table 3:** Changes in MSAP profiles of hybrids #60 and #105 between 2010 and 2013.

MSAP profile	Hybrid #60		Hybrid #105	
No change over time	239	94.1%	265	92.3%
Methylations	2	0.8%	2	0.7%
Demethylations	3	1.8%	6	2.1%
Ambiguous	10	3.9%	14	4.9%

### Sequence analysis

Out of the total MSAP polymorphic bands derived from the drought stress treatments, 43 could be cloned and sequenced. For the others, technical limitations such as the close proximity among bands, the lack of amplification or amplification of more than one band when using the MSAP selective primers impeded the acquisition of the whole sequences.

The identity of the sequenced polymorphic MSAP bands was first assessed through BLASTX analysis against the non-redundant protein database and BLASTN analysis against the nucleotide database, allowing the annotation of eleven sequences: ATP-dependent DNA helicase 2 subunit KU80, BTB/POZ and MATH domain-containing protein 1-like, Transcription initiation factor TFIID subunit 2, gibberellin 2-β-dioxygenase 1-like, WEB family protein At2g38370-like, an rRNA gene, a centromeric retrotranposon, two hypothetical proteins, and two putative transposons (S3 Table). Taken together, the results obtained from BLASTX and BLASTN analysis suggest that 8 out of the 43 (19%) sequenced bands are gene-related sequences while 3 out of 43 (7%) are related to transposable elements. Although several of the annotations correspond to hypothetical proteins, and thus provide no information concerning the function of these genes, the relevance of their identification resides in the fact that they appear to come from expressed genome regions, i.e., genes. Additionally, a high proportion of the methylation polymorphisms (74%) did not show sequence similarity to coding regions, demonstrating a high rate of methylation and demethylation in non-coding or non-sequenced regions, underscoring the need for the complete genome sequences of this species.

Our data show strong similarities to previous MSAP studies on the UNST1131 plant, which was obtained by chromosome doubling in a seed of the diploid plant UNST1122, as some of the polymorphisms identified had similarity to repetitive sequences [22]. Moreover, sequences with similarity to transposable elements have been shown to be differentially expressed in sexual and apomictic *E*. *curvula* genotypes [43]. More recently, a Copia-10_like element was reported to be expressed in the inflorescences of sexual genotypes, but not of apomictic ones, suggesting that transposable elements activation in sexual genotypes could be associated with mechanisms related to the expression of sexuality [43].

As mentioned previously, transposon movement has been reported following different stresses in several species, including rice [44] and wheat [45]. The identification of transposable elements among the MSAP fragments could indicate that stress had modulated their transcriptional activity and thereby affected the frequency of sexual reproduction. Transposable elements and the epigenetic machinery have already been proposed as key players in fostering phenotypic and biological innovations during major ecological transitions [46]. TEs and the epigenetic component are sensitive to several global change stressors and are triggering genomic and phenotypic responses to these stressors [47]. These mechanisms can be operating within the transition from apomixis to sexuality in facultative apomicts.

## Conclusions

Throughout their lives, plants face changing environmental situations that inevitably cause stress. Plants have developed strategies that allow them to survive and reproduce during these stressful periods. Among these strategies new discoveries point out to transposable elements and epigenetic components as important players in these processes. Recently, Rey et al. [47] have proposed an integrative molecular engine coupling both elements that allow organisms to fine-tune phenotypes in a real-time fashion, adjust the production of phenotypic and genetic variation, and produce heritable phenotypes with different levels of transmission fidelity.

Generating genetic variability in order to cope with changing environmental conditions is crucial for a species survival. Under specific conditions, the ratio between sexual and asexual embryo sacs may further vary among genotypes, indicating that the mode of reproduction in facultative asexual plants is often determined by genotype-by-environment interactions [48]. The increase in sexual reproduction under stress conditions agrees with the oxidative stress initiation hypothesis [48] postulating that oxidative stress could activate meiosis-specific proteins that initiate double strand break formation and thus increase recombination frequency, suggesting that meiosis might represent a cellular survival strategy.

From the results presented here, we conclude that different stress situations, both exogenous and endogenous, including drought, tissue culture, and hybridization can alter the ratio of apomictic/sexual embryo sacs in facultative tetraploid cultivars of *E*. *curvula*. These changes occur together with genetic and epigenetic changes, primarily in methylation affecting both coding and non-coding regions and involving repetitive elements [22]. These data together with previous observations [20, 21, 49, 50, 51, 52, 53, 54] allow us to generate a hypothetical model of the regulation of apomixis in weeping lovegrass in which the genetic/s region/s that condition apomixis, is/are affected by ploidy, and is/are subjected to epigenetic control. This region would be present in the tetraploid apomictic plants and, most likely, in diploid where it is not expressed. Support for this hypothesis comes from Cardone et al [50], who demonstrated that the apomictic region is present, but repressed, at the diploid level. This region might be absent in the sexual tetraploid (OTA-S), which never produces apomictic embryo sacs.

The apomixis-conditioning region was mapped in *Pennisetum* and *Paspalum* [55, 56]. In *Paspalum notatum*, this apo-locus is a region of low recombination and abundant repetitive elements that likely contains duplicate genes [57]. Duplicated genomic regions can cause silencing, as is often the case for regions containing transgenes [58]. A similar process occurs in genomic regions containing transposons in order to maintain the transposons in a silenced state [59]. Taking into account this information and our results, we consider it likely that the apo region contains genes that are duplicated in another genomic region that codify for the sexual development. The presence of extra copies of these genes in apomictic plants could induce silencing of the genes responsible from sexual development. TEs could also be involved. Thus, we propose that an epigenetic mechanism could silence this region during apomictic development and that stress conditions may disturb or interrupt this silencing signal, allowing for a switch to sexual reproduction until the stress is no longer present, at which point the network is restored and sexual reproduction is once more silenced.

## Supporting information

S1 Table Primers and adapters used in the RAPD, AFLP and MSAP experiments. (DOCX)

S2 Table MSAP pattern analysis. The first and the second four columns show the polymorphic band pattern in an acrylamide gel.
(DOCX)

S3 Table Polymorphisms analysis. Polymorphisms in time in plants of cv. Tanganyika INTA treated by drought stress and after stress ceased (MSAP) and hybrid #105 at the time of obtaining and after three years (AFLP).
(DOCX)
