# Supplementary material for: Apomixis frequency under stress conditions in weeping lovegrass (Eragrostis curvula)
Source: PLoS One. 2017 Apr 18;12(4):e0175852. doi: 10.1371/journal.pone.0175852 (PMC5395188; doi:10.1371/journal.pone.0175852)
Supplement: S1 Table — (DOCX) [file pone.0175852.s001.docx]

**S1 Table.** **Primers and adapters used in the RAPD, AFLP and MSAP experiments.**

| **Name** | **Experiment** | **Sequence (5’-3’)** |
| --- | --- | --- |
| 232  245  304  331  *Pst*I adapter upper | RAPD  RAPD  RAPD  RAPD  AFLP | CGG TGA CAT C  CGC GTG CCA G  AGT CCT CGC C  CTA GAG GCC G  GAC TGC GTA GGT GCA |
| *Pst*I adapter lower | AFLP | CCT ACG CAG TCT ACG AG |
| *Mse*I adapter upper | AFLP | GAC GAT GAG TCC TGA G |
| *Mse*I adapter lower | AFLP | ATG AGT CCT GAG TA |
| *Pst*I + 1 | AFLP | GAC TGC GTA CAT GCA GA |
| *Pst*I + 3 P36 | AFLP | GAC TGC GTA CAT GCA GACC |
| *Pst*I + 3 P37 | AFLP | GAC TGC GTA CAT GCA GACG |
| *Pst*I + 3 P40 | AFLP | GAC TGC GTA CCA ATT CAG T |
| *Mse*I + 1 | AFLP | GAT GAG TCC TGA GTA AA |
| *Mse*I + 3 M31 | AFLP | GAT GAG TCC TGA GTA AAAA |
| *Mse*I + 3 M38 | AFLP | GAT GAG TCC TGA GTA AACT |
| *Mse*I + 3 M39 | AFLP | GAT GAG TCC TGA GTA AAGA |
| *Mse*I + 3 M43 | AFLP | GAT GAG TCC TGA GTA AATA |
| *Mse*I + 3 M45 | AFLP | GAT GAG TCC TGA GTA AATG |
| *Hpa*II-*Msp*I adapter upper | MSAP | GACGATGAGTCTAGAA |
| *Hpa*II-*Msp*I adapter lower | MSAP | CGTTCTAGACTCATC |
| *Eco*RI + 1 | MSAP | GACGGCGTACCAATTCA |
| *Eco*RI + 3 E32 | MSAP | GACGGCGTACCAATTCAAC |
| *Eco*RI + 3 E34 | MSAP | GACGGCGTACCAATTCAAT |
| *Eco*RI + 3 E35 | MSAP | GACGGCGTACCAATTCACA |
| *Eco*RI + 3 E36 | MSAP | GACGGCGTACCAATTCACC |
| *Eco*RI + 3 E37 | MSAP | GACGGCGTACCAATTCACG |
| *Eco*RI + 3 E40 | MSAP | GACGGCGTACCAATTCAGC |
| *Eco*RI + 3 E41 | MSAP | GACGGCGTACCAATTCACT |
| *Eco*RI + 3 E42 | MSAP | GACGGCGTACCAATTCCAC |
| *Eco*RI + 3 E43 | MSAP | GACGGCGTACCAATTCCGG |
| *Hpa*II-*Msp*I + 0 | MSAP | GATGAGTCTAGAACGG |
| *Hpa*II-*Msp*I + 3 HM2 | MSAP | GATGAGTCTAGAACGGAAT |
| *Hpa*II-*Msp*I + 3 HM3 | MSAP | GATGAGTCTAGAACGGATC |
| *Hpa*II-*Msp*I + 3 HM4 | MSAP | GATGAGTCTAGAACGGAGT |
| *Hpa*II-*Msp*I + 3 HM5 | MSAP | GATGAGTCTAGAACGGAGC |
| *Hpa*II-*Msp*I + 3 HM6 | MSAP | GATGAGTCTAGAACGGACA |
| *Hpa*II-*Msp*I + 3 HM7 | MSAP | GATGAGTCTAGAACGGACT |
| *Hpa*II-*Msp*I + 3 HM8 | MSAP | GATGAGTCTAGAACGGTGA |
| *Hpa*II-*Msp*I + 3 HM9 | MSAP | GATGAGTCTAGAACGGTAC |
| *Hpa*II-*Msp*I + 3 HM10 | MSAP | GATGAGTCTAGAACGGTGC |
| *Hpa*II-*Msp*I + 3 HM11 | MSAP | GATGAGTCTAGAACGGTCT |
