# Supplementary material for: Apomixis frequency under stress conditions in weeping lovegrass (Eragrostis curvula)
Source: PLoS One. 2017 Apr 18;12(4):e0175852. doi: 10.1371/journal.pone.0175852 (PMC5395188; doi:10.1371/journal.pone.0175852)
Supplement: S2 Table — The first and the second four columns show the polymorphic band pattern in an acrylamide gel. (DOCX) [file pone.0175852.s002.docx]

**S2 Table.** **MSAP pattern analysis.** The first and the second four columns show the polymorphic band pattern in an acrylamide gel.

| **Polymorphic bands** | | | | **Pattern** | | | |
| --- | --- | --- | --- | --- | --- | --- | --- |
| *Hpa*II | *Msp*I | *Hpa*II | *Msp*I | *Hpa*II | *Msp*I | *Hpa*II | *Msp*I |
| Before | | After | | Before | | After | |
| **De-methylation events** | | | | | | | |
|  |  |  |  | 0 | 1 | 1 | 1 |
|  |  |  |  | 0 | 1 | 1 | 0 |
|  |  |  |  | 1 | 1 | 1 | 0 |
| **Methylation events** | | | | | | | |
|  |  |  |  | 1 | 1 | 0 | 1 |
|  |  |  |  | 1 | 0 | 1 | 1 |
|  |  |  |  | 1 | 0 | 0 | 1 |
| **Ambiguous polymorphisms** | | | | | | | |
|  |  |  |  | 0 | 0 | 0 | 1 |
|  |  |  |  | 0 | 0 | 1 | 1 |
|  |  |  |  | 0 | 0 | 1 | 0 |
|  |  |  |  | 1 | 1 | 0 | 0 |
|  |  |  |  | 0 | 1 | 0 | 0 |
|  |  |  |  | 1 | 0 | 0 | 0 |
