# Supplementary material for: Apomixis frequency under stress conditions in weeping lovegrass (Eragrostis curvula)
Source: PLoS One. 2017 Apr 18;12(4):e0175852. doi: 10.1371/journal.pone.0175852 (PMC5395188; doi:10.1371/journal.pone.0175852)
Supplement: S3 Table — Polymorphisms in time in plants of cv. Tanganyika INTA treated by drought stress and after stress ceased (MSAP) and hybrid #105 at the time of obtaining and after three years (AFLP). (DOCX) [file pone.0175852.s003.docx]

**S3 Table.** **Polymorphisms analysis.** Polymorphisms in time in plants of cv. Tanganyika INTA treated by drought stress and after stress ceased (MSAP) and hybrid #105 at the time of obtaining and after three years (AFLP).

| Polymor-phism # | GenBank  Accession | Length (bp) | BLAST | | | | | | |
| --- | --- | --- | --- | --- | --- | --- | --- | --- | --- |
|  |  |  | Description | GenBank  database | Accession no. | Score | E-value | Identity (%) | Query coverage (%) |
| MP#2 | KS518265 | 121 | *Oryza sativa* Japonica Group Os09g0324400 mRNA, complete cds | nt | NM_001069412.1 | 89.7 | 9e ^-15^ | 83 | 78 |
| MP#2 | KS518265 |  | hypothetical protein F775_42941 [*Aegilops tauschii*] | nr | EMT26398.1 | 49.3 | 8e ^-6^ | 70 | 74 |
| MP#4 | KS518266 | 244 | transposon protein, putative, unclassified [*Oryza sativa* Japonica Group] | nr | AAX96213.1 | 66.6 | 2e ^-10^ | 82 | 41 |
| MP#5 | KS518267 | 522 | Predicted: ATP-dependent DNA helicase 2 subunit KU80 [*Setaria italica*] | nr | ACN25700.1 | 61.2 | 8e ^-8^ | 53 | 54 |
| MP#6 | KS518268 | 283 | Predicted: BTB/POZ and MATH domain-containing protein 1-like [*Setaria italica*] | nr | XP_004958935.1 | 129 | 2e ^-33^ | 72 | 87 |
| MP#7 | KS518269 | 397 | *Sorghum bicolor* hypothetical protein, mRNA | nt | XM_002466717.1 | 156 | 7e ^-35^ | 71 | 92 |
| MP#7 | KS518269 |  | hypothetical protein SORBIDRAFT_01g013650 [*Sorghum bicolor*] | nr | XP_002466762.1 | 107 | 1e ^-24^ | 62 | 84 |
| MP#9 | KS518270 | 301 | *Zea mays* gibberellin 2-beta-dioxygenase (LOC100284370), mRNA | nt | NM_001157265.1 | 87.8 | 3e ^-14^ | 88 | 97 |
| MP#9 | KS518270 |  | Predicted: gibberellin 2-beta-dioxygenase 1-like [*Setaria italica*] | nr | XP_004968599.1 | 50.8 | 5e ^-5^ | 87 | 22 |
| MP#10 | KS518274 | 266 | *Setaria italica* WEB family protein At2g38370-like (LOC101779700), mRNA | nt | XM_004961700.2 | 302 | 8e ^-79^ | 85 | 99 |
| MP#14 | KS518271 | 158 | Transcription initiation factor TFIID subunit 2 [*Aegilops tauschii*] | nr | EMT28831.1 | 68.2 | 2e ^-11^ | 91 | 60 |
| MP#21 |  | 184 | *Lecomtella madagascariensis* 18S rRNA gene, ITS1, 5.8S rRNA gene, ITS2 and 26S rRNA gene, specimen voucher K:MSV603 | nt | HG315108.1 | 333 | 1e ^-87^ | 100 | 100 |
| MP#22 | KS518272 | 314 | retrotransposon protein, putative, unclassified [*Oryza sativa* Japonica Group] | nr | ABA98491.1 | 100 | 9e ^-22^ | 50 | 85 |
| MP#23 | KS518273 | 310 | *Zea mays* CRM centromeric retrotranposon, complete sequence | nt | AY129008.1 | 268 | 1e ^-87^ | 83 | 83 |
